# Mechanisms, Mediators, and Moderators of the Effects of Exercise on Chemotherapy-Induced Peripheral Neuropathy

**DOI:** 10.3390/cancers14051224

**Published:** 2022-02-26

**Authors:** Kaitlin H. Chung, Susanna B. Park, Fiona Streckmann, Joachim Wiskemann, Nimish Mohile, Amber S. Kleckner, Luana Colloca, Susan G. Dorsey, Ian R. Kleckner

**Affiliations:** 1 Department of Surgery, Wilmot Cancer Institute, University of Rochester Medical Center, 265 Crittenden Blvd., Box CU 420658, Rochester, NY 14642, USA; kaitlinchung2004@gmail.com (K.H.C.); amber.kleckner@umaryland.edu (A.S.K.); 2 Faculty of Medicine and Health, School of Medical Sciences, Brain and Mind Centre, The University of Sydney, Sydney, NSW 2006, Australia; susanna.park@sydney.edu.au; 3 Department of Sport, Exercise and Health, University of Basel, 4052 Basel, Switzerland; fiona.streckmann@unibas.ch; 4 Department of Oncology, University Hospital Basel, 4031 Basel, Switzerland; 5 Department of Medical Oncology, National Center for Tumor Diseases and Heidelberg University Hospital, 69120 Heidelberg, Germany; joachim.wiskemann@nct-heidelberg.de; 6 Department of Neurology, University of Rochester Medical Center, Rochester, NY 14642, USA; nimish_mohile@urmc.rochester.edu; 7 Department of Pain and Translational Symptom Science, University of Maryland School of Nursing, Baltimore, MD 21201, USA; colloca@umaryland.edu (L.C.); sdorsey@umaryland.edu (S.G.D.); 8 Center to Advance Chronic Pain Research (CACPR), University of Maryland, Baltimore, MD 21201, USA

**Keywords:** CIPN, biomarker, neurotoxicity, optimization, brain, mitochondria

## Abstract

**Simple Summary:**

Chemotherapy-induced peripheral neuropathy (CIPN) is a common side effect of cancer treatment. It is experienced as numbness, tingling, pain, and cramping in the hands and/or feet and can interfere with daily living. Exercise is a promising treatment for CIPN but its underlying mechanisms are understudied. Herein, we discuss potential mechanisms underlying how exercise might treat CIPN (e.g., anti-inflammatory cytokines, self-efficacy, and social support) and what demographic (e.g., age and sex) and clinical characteristics (e.g., body mass index), and exercise routines (e.g., timing, intensity, and type of exercise) may moderate the effects of exercise on relieving CIPN. These details can help clinicians predict who will get CIPN, enable clinicians to tailor exercise programs to patients based on specific characteristics, and inform future research and biomarkers on the relationship between exercise and CIPN.

**Abstract:**

Chemotherapy-induced peripheral neuropathy (CIPN) is an adverse effect of neurotoxic antineoplastic agents commonly used to treat cancer. Patients with CIPN experience debilitating signs and symptoms, such as combinations of tingling, numbness, pain, and cramping in the hands and feet that inhibit their daily function. Among the limited prevention and treatment options for CIPN, exercise has emerged as a promising new intervention that has been investigated in approximately two dozen clinical trials to date. As additional studies test and suggest the efficacy of exercise in treating CIPN, it is becoming more critical to develop mechanistic understanding of the effects of exercise in order to tailor it to best treat CIPN symptoms and identify who will benefit most. To address the current lack of clarity around the effect of exercise on CIPN, we reviewed the key potential mechanisms (e.g., neurophysiological and psychosocial factors), mediators (e.g., anti-inflammatory cytokines, self-efficacy, and social support), and moderators (e.g., age, sex, body mass index, physical fitness, exercise dose, exercise adherence, and timing of exercise) that may illuminate the relationship between exercise and CIPN improvement. Our review is based on the studies that tested the use of exercise for patients with CIPN, patients with other types of neuropathies, and healthy adults. The discussion presented herein may be used to (1) guide oncologists in predicting which symptoms are best targeted by specific exercise programs, (2) enable clinicians to tailor exercise prescriptions to patients based on specific characteristics, and (3) inform future research and biomarkers on the relationship between exercise and CIPN.

## 1. Introduction

Chemotherapy-induced peripheral neuropathy (CIPN) is a severe, dose-limiting effect of neurotoxic antineoplastic agents utilized to treat common solid tumors and hematologic cancers [1,2]. Two thirds of patients who undergo taxane, vinca alkaloid, platinum agent, proteasome inhibitors, or thalidomide-based chemotherapeutic regimens develop CIPN [3]. While CIPN can be an acute condition present in the hours and days after a treatment infusion [3], approximately 58–78% of patients still experience symptoms one month following the completion of chemotherapy [3], and many endure CIPN for years [4,5].

Symptoms of CIPN primarily manifest as deficits in sensory and motor functions [6]. Sensory and motor dysfunctions may include numbness, tingling, hypersensitivity to cold temperatures (e.g., touching a cold object or drinking a cold beverage) [2,7], hyperalgesia in the hands and feet, cramping, distal extremity weaknesses, and balance problems (i.e., postural instability) [8,9] resulting in increased risk of falling [10,11]. These symptoms can reduce patients’ quality of life by inhibiting their ability to perform daily activities such as walking, dressing, and typing [6]. By altering medical therapy and negatively impacting cancer progression, these symptoms can also influence patients’ clinical outcomes and survival [6,7]. Severity and symptom profiles of CIPN differ between various classes of systemic therapy [3], suggesting potentially distinct etiology and mechanisms producing CIPN symptoms.

Despite over 20 years of research and over 100 clinical trials [12,13], duloxetine is the only intervention recommended to treat CIPN. Amid highly limited medication-based treatment options, exercise has emerged as a promising and safe intervention for CIPN, as suggested by approximately two dozen randomized and non-randomized clinical trials to date [14,15,16]. This is in line with a large body of research suggesting that exercise improves other common cancer-related health outcomes, including fatigue, anxiety, depression, physical functioning, and health-related quality of life [17]. Our systematic review of exercise and CIPN last year identified 15 randomized controlled trials (RCTs) of resistance, balance, aerobic, or multimodal exercise training vs. non-exercise control on various CIPN signs or symptoms. Six of these studies reported benefits as the study’s primary outcome, eight studies reported benefits as a secondary outcome, five studies reported no significant effect of exercise, and no studies reported exercise was worse than the non-exercise control condition. Moreover, a recent meta-analysis across seven RCTs found that in patients with cancer at risk for CIPN, exercise yielded beneficial effects on static balance (overall effect size (ES) = 0.46) and neuropathic symptoms (overall ES = 0.43) [18], reinforcing the promise of exercise for CIPN yet the need for future definitive studies. Research on exercise and CIPN is also a rapidly growing area of research—our recent review found that 12 of the 23 identified studies were published from 2019 to 2021 with 19 more studies preregistered [15].

As additional trials test whether exercise treats CIPN, it becomes increasingly important to develop mechanistic understanding of the effects of exercise [19] to determine how it can be (1) optimized to best target CIPN symptoms (i.e., identify mediators) and (2) tailored for patients who are most prone to respond (i.e., identify moderators). CIPN is a heterogenous condition and the effect of exercise on CIPN differs across individuals [20,21]. Therefore, it is likely that exercise will be optimal when prescribed for patients who are prone to benefit, as has been suggested by a recent study where baseline neurophysiological testing predicted both patient postural control profiles and the extent of benefit of balance exercises on CIPN [22]. Although there is significant evidence suggesting benefits of exercise for CIPN [14,15], the mechanisms of these effects remain unclear, and they are rarely reviewed. Thus, our goal was to prepare the most comprehensive review of potential mechanisms, mediators, moderators, and biomarkers of the effects of exercise on CIPN to date.

**Figure 1:**
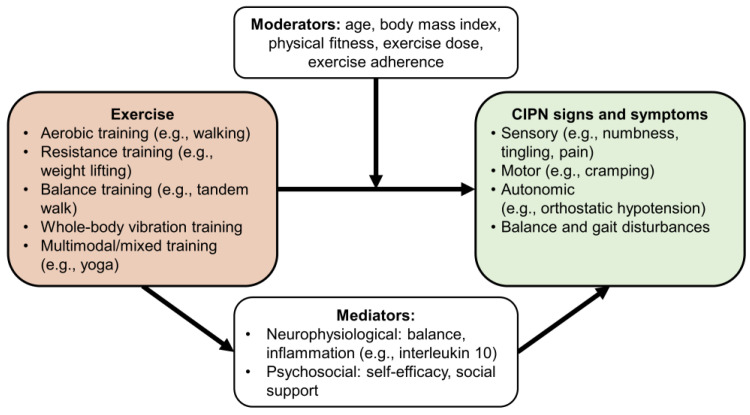
This model illustrates the different ways in which mediators and moderators impact the effect of exercise on chemotherapy-induced peripheral neuropathy (CIPN) symptoms. Mediators are factors on the causal pathway between exercise and CIPN symptoms (i.e., factors that determine how exercise exerts its effects), while moderators are factors that affect the magnitude of the effect of exercise on CIPN (i.e., factors that determine how large the effect of exercise is). Mediators can be used to tailor and optimize exercise for individual patients, and moderators can be used to predict who will respond best to exercise.

To begin to illuminate the relationship between exercise and CIPN improvement, we conducted a narrative review of evidence for the (1) mechanisms that explain the relationship between exercise and CIPN (e.g., neurophysiological and psychosocial factors), (2) mediators—factors along the causal pathway between exercise and CIPN symptoms (i.e., how exercise exerts its effects [23]; e.g., anti-inflammatory cytokines, self-efficacy, social support), and (3) moderators—factors that affect the magnitude of the effect of exercise on CIPN (i.e., how large the effect of exercise is [23]; e.g., age, sex, body mass index, physical fitness, exercise dose (duration and intensity of exercise), exercise adherence, and timing of exercise (prior to, during, or after chemotherapy; Figure 1). For this narrative review, a literature search of PubMed and Google Scholar was conducted for studies that (1) tested a behavioral intervention to treat patients affected by cancer, CIPN, or CIPN-related conditions and (2) included measures, analysis, and/or discussion of mediating and/or moderating variables. Studies were examined to determine the relevance of their findings in the context of mechanistic understanding of the effects of exercise on CIPN.

The knowledge reviewed and synthesized herein can contribute to (1) guiding oncologists in predicting which symptoms are best targeted by specific exercise programs, (2) enabling clinicians to tailor exercise prescriptions to patients based on specific characteristics, and (3) informing future research and biomarkers on the relationship between exercise and CIPN for both preclinical and clinical studies. In the future, knowledge of mechanisms, mediators, and moderators could help tailor the treatment of CIPN to the needs of each single patient in line with precision medicine initiatives in exercise oncology [24] and exercise science more broadly [25].

## 2. Neurophysiological Mechanisms

**Figure 2:**
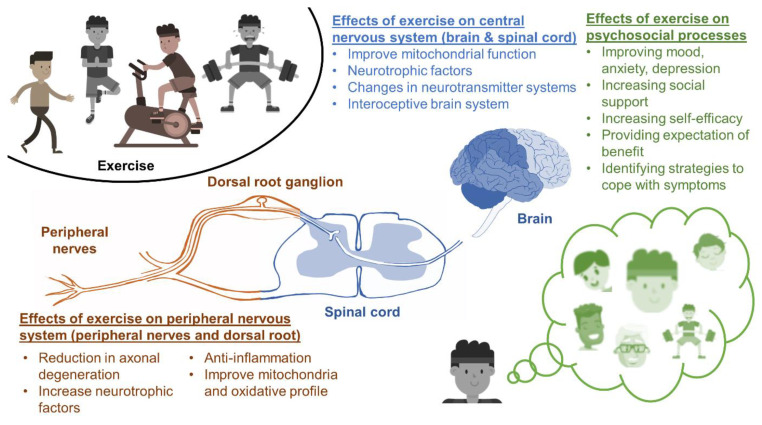
Exercise may treat or prevent CIPN through effects on the peripheral nervous system, the central nervous system, and psychosocial processes. This figure was reproduced with changes from Kleckner et al., 2021 with the permission of Springer Nature Publishing [16].

Exercise has been suggested to alleviate neuropathy through various neurophysiological mechanisms in the peripheral nervous system, in the central nervous system, and via psychosocial processes [26] (Figure 2). These mechanisms work synergistically at molecular, subcellular, cellular, and neural levels to improve peripheral nerve function, as shown by studies modeling traumatic peripheral nerve injury with humans and mice [27]. Although experimental data specifically relating to mechanisms of exercise-induced benefit on CIPN is limited, the key mechanisms underlying the effects of exercise on peripheral nerve function more generally are likely applicable to CIPN [15].

### 2.1. Neurotrophic Factors

CIPN involves damage to peripheral nerve axons, which causes nerve dysfunction that ranges from acute sensations to permanent damage and chronic pain [6]. Exercise has been shown to enhance the expression of neurotrophic factors (i.e., proteins that support the survival, development and function of neurons), specifically the glial cell line-derived growth factor (GDNF), brain-derived neurotrophic factor (BDNF), and insulin-like growth factor 1 (IGF-1) [28]. In a study assessing the relationship between treadmill use and neurotrophic factors, GDNF, BDNF, and IGF-1 were elevated in the blood, nerves, and muscles of exercising animals [28]. However, a separate study found that although treadmill training enhanced axonal regeneration, it could not improve regeneration in mice without sources of BDNF in both axonal and Schwann cells, which underscores the importance of neurotrophic factors [29]. Nevertheless, it is important to note that upregulation of neurotrophic factors is not solely associated with regeneration and may also be correlated with neuropathic pain and its maintenance [30]. Accordingly, it will be important to closely examine the impact of exercise on neurotrophic factors in the clinical setting to clarify their role in neuropathic pain mitigation and treatment.

### 2.2. Inflammation

Proinflammatory cytokine upregulation and inflammatory cascade activation are implicated in the development of CIPN [31,32]. Because exercise has been shown to have anti-inflammatory effects, it may have the potential to ameliorate CIPN via anti-inflammatory cascades. Exercise reduces inflammation and oxidative stress in the body by promoting the release of anti-inflammatory cytokines and reducing levels of oxidative markers [33]. Contracting muscles during exercise release proinflammatory IL-6 [34], which causes an increase in anti-inflammatory IL-10 and IL-1RA [35]. Moreover, moderate-intensity aerobic and resistance training during chemotherapy have been suggested to improve markers of inflammation by strengthening correlations between IL-10 and IL-6 and reducing IFNγ and IL-1β [36].

### 2.3. CIPN and the Brain

Brain activity is a key element in the construction of all mental states [37], including CIPN symptoms. In response to heat-induced pain on the leg, patients with CIPN have exhibited brain hyperactivity in sensory regions (e.g., insula) and in the posterior part of the default mode network, specifically the ventral precuneus [38]. Studies have suggested that this hyperactivity in the brain, particularly in the insula, is positively correlated with CIPN severity and that a reduction of brain activity in the insula is associated with a reduction of CIPN symptom severity [39]. This makes sense because the insula is a hub in the brain that supports interoception [40], which is the processing of bodily sensations [41], including sensations related to CIPN. Our recent review of the role of the brain in CIPN implicated several pathways potentially underlying or related to brain hyperactivity, including reduced GABAergic inhibition, neuroinflammation, and overactivation of GPCR/MAPK pathways, and implicating a role for interoception in CIPN [39]. There are no published studies investigating the effects of exercise on both CIPN and the brain, but our preliminary work suggests a role for exercise reducing functional connectivity in the interoceptive brain system, particularly rooted connections with the posterior cingulate cortex [42,43]. Therefore, tracking brain activity and connectivity with non-invasive tools such as functional magnetic resonance imaging could be a useful biomarker for learning more about the effects of exercise on the brain in CIPN.

### 2.4. Mitochondrial Function

Mitochondrial dysfunction is also implicated in the etiology of CIPN [44,45,46]. Because exercise may have a large effect on the regulation of mitochondrial bioenergetics, exercise has the potential to mitigate the onset and progression of CIPN. Mitochondria are dynamic cellular organelles that are responsible for ATP energy production. To efficiently generate energy, mitochondria rely on robust oxidative phosphorylation machinery; appropriate levels of reactive oxygen species; and efficient transport and compartmentalization of nutrients, calcium, and other ions. To maintain stable high levels of functioning, sufficient rates of both fusion and fission as well as mitophagy (degradation of malfunctioning mitochondria) and biogenesis (production of new mitochondria) are necessary. Vincristine, paclitaxel, cisplatin, oxaliplatin, and bortezomib all cause mitochondrial pathophysiology in neurons, glial cells, and other cell types pertinent to CIPN [44,46]. For example, paclitaxel, vincristine, and bortezomib can alter calcium homeostasis in the mitochondria, which can interfere with calcium-dependent release of neurotransmitters [44]. In addition, cisplatin and oxaliplatin chemotherapy induce adducts between the platinum ion and mitochondrial DNA (mtDNA) [47]. While nuclear DNA can be repaired by base excision repair and nucleotide excision repair pathways, mtDNA is not privy to these processes, thereby thwarting mtDNA gene expression and replication.

Exercise has strong pleiotropic effects on mitochondrial health in many cell types, including neurons and non-neuronal cells in the central and peripheral nervous systems [48]. Exercise increases mitochondrial antioxidant capacity, electron transport chain efficiency, and biogenesis and decreases oxidative stress and mtDNA damage [49]. Specifically, exercise promoted neurogenesis and hippocampal plasticity via improvements in mitochondrial function in a mouse model [50]. Furthermore, exercise is touted to attenuate age-related cognitive decline and prevent neurodegenerative disorders in part due to its ability to increase the mitochondrial fortitude and resistance to stress [51]. Marques-Aleixo et al., 2016 assessed the effects of endurance exercise (a 12-week treadmill program or voluntary free wheel running) on doxorubicin-induced mitochondrial dysfunction in a rat model [52]. Both exercise modalities attenuated doxorubicin-induced behavioral alterations (i.e., Y-maze and open field tests, which assess exploratory behavior), though they did not assess CIPN-specific signs. In this same study, exercise protected against the doxorubicin-induced opening of the mitochondrial permeability transition pore, reduction in mitochondrial biogenesis, and accumulation of reactive oxygen species [52]. To our knowledge, there have not yet been any studies testing the hypothesis that exercise can attenuate chemotherapy-induced mitochondrial pathophysiology and, as a result, prevent the onset or progression of CIPN. However, mitochondrial mechanisms underlying CIPN in humans and animal models is a promising avenue for future research.

### 2.5. Axonal Integrity and Outgrowth

As shown in mouse models, exercise can promote axonal outgrowth and protect against axonal degeneration, a key pathological feature of CIPN. In a mouse model of paclitaxel-induced neurotoxicity, for example, treadmill exercise was shown to protect against paclitaxel-induced axonal degeneration [27], preventing reduction in intraepidermal nerve fiber (IENF) density and partially ameliorating reductions in sensory caudal nerve amplitude [27]. Separate studies examining treadmill training in mice with nerve injury resulted in enhanced axonal sprouting and regeneration [53], improved functional recovery, increased number of myelinated fibers [54], and reduced allodynia [55].

Exercise has also been demonstrated to improve IENF regeneration in clinical studies. A 12-month weekly supervised exercise program in patients with diabetes and no neuropathy resulted in improved IENF density [56]. A moderate-intensity 10-week exercise program in patients with diabetic neuropathy also produced improved IENF branching at proximal biopsy sites, although there were no changes in IENF density, sensory, or motor nerve conduction studies [57]. However, there have been no studies of exercise in CIPN patients that have examined IENF density.

Only a few exercise and CIPN studies have involved assessment of nerve conduction, none of which have identified improvement post-intervention on nerve conduction studies. A 20-week sensorimotor and resistance training program in patients with cancer undergoing chemotherapy included nerve conduction studies as part of a neurological grading scale. The results revealed that exercise did not have an impact on nerve conduction between the groups [58].

## 3. Psychosocial Mechanisms

Current understanding of the psychosocial mechanisms behind the impact of exercise on CIPN is limited, and is primarily based on the relationship between exercise and mental health (e.g., mood, well-being, anxiety, and depression), which is related to CIPN [32,59]. Despite the fact that the psychosocial benefits of exercise are well-established, we believe it is prudent to include this discussion to (1) emphasize the importance of these mechanisms in the specific context of CIPN, (2) bridge cross-disciplinary knowledge gaps across CIPN research, and (3) promote future research in this area.

### 3.1. Improving Mood, Anxiety, Depression, and Fatigue

CIPN symptoms can limit activities of daily living and contribute to fatigue, anxiety, and depression [32,59,60]. Exercise may treat CIPN through known mechanisms for improving mood and reducing distress. For example, moderate-intensity aerobic exercise (e.g., walking) can offer time away from stress, expose patients to greenspace, and provide opportunity for socialization [61,62]. Meta-analytical studies suggest that this kind of exercise can have a positive anxiolytic and depression-reducing effect, potentially involving neurological mechanisms such as the interoceptive brain system [32,63]. In addition, aerobic, resistance, and anaerobic exercise have shown efficacy in improving cancer-related fatigue during and after primary treatment, possibly by way of improving oxygen circulation in the body and increasing energy efficiency [32,64].

### 3.2. Increasing Social Support and Self-Efficacy

Exercise has been shown to boost social support and self-efficacy (i.e., the belief that one can accomplish a specific goal [65,66,67]). Group or partner exercise is associated with increased social support and exercise adherence [68,69]. This can create a positive feedback loop that may reduce distress associated with CIPN. Conjointly, improvements in self-efficacy can help a patient reconceptualize personal perceptions of cancer treatment and CIPN symptoms as a challenge rather than a threat (i.e., the idea that patients have sufficient resources to surmount the stressful situation). Compared to identical situations that are perceived as challenges, situations perceived as threats tend to increase blood pressure and cortisol levels [70]. A challenge-oriented mindset may help reduce psychological stress and inflammation, thereby reducing CIPN symptoms.

### 3.3. Expectation of Benefit

The theory that exercise may cause patients to expect certain benefits has been explored in sports medicine through placebo effects [71,72]. The placebo effect is a valid and potentially valuable psychosocial-level mechanism for treating patients with CIPN [72,73]. Patient expectations for the benefits of exercise can by affected by (1) who introduces exercise (e.g., a figure of authority—an exercise physiologist, medical doctor, or physical therapist); (2) how exercise is introduced (i.e., whether it is framed as definitively beneficial or potentially beneficial); (3) where exercise is conducted (e.g., at a state-of-the-art exercise facility); (4) the patient’s prior experience with exercise; and (5) the patient’s social context (e.g., having relatives, peers, or role models who advocate for exercise) [72]. Future studies of exercise and CIPN should assess outcome expectations (e.g., how much patients expect to improve) and treatment expectations (e.g., which exercise treatment patients believe may work best) to better understand the role of expectations in facilitating exercise-related benefits.

## 4. Mediators and Moderators

### 4.1. Mediators of the Effects of Exercise on CIPN

Although studies of mediators have been performed for other cancer-related symptoms, no studies have specifically explored the mediators of the effects of exercise on CIPN. Therefore, we speculate as to how mediators in the existing studies may apply to CIPN in service of informing future study designs.

One commonly suggested neurophysiological mediator of exercise and CIPN is inflammation. Because inflammation has been implicated in CIPN [31,32] and exercise has been shown to have anti-inflammatory effects [33,36], exercise is a promising treatment for inflammation-related CIPN. If a patient is known to have high levels of inflammation, then it will likely be beneficial to prescribe an exercise program that is targeted towards anti-inflammation. For instance, moderate-intensity aerobic and resistance training during chemotherapy have been suggested to improve markers of inflammation by strengthening correlations between IL-10 and IL-6 and reducing IFNγ and IL-1β [36]. Exercise also bolsters the immune system, which improves overall immunity and influences the release of cytokines and other immune modulators [74].

Fatigue is another potential mediator of the effects of exercise on CIPN. Lin et al., 2019 introduced a four-week yoga therapy program for cancer survivors (*n* = 410) that examined the mediating effects of yoga-related changes in sleep on cancer-related fatigue [75]. This exploratory study suggested that yoga improved sleep-mediated changes in fatigue by improving daytime dysfunction and sleep quality [75]. At a psychosocial level, yoga could have benefited patients by offering time away from stress, increasing energy levels, encouraging more activity throughout the day, and reducing sleep inertia [75]. It is plausible that similar mechanisms could also apply to CIPN. First, yoga has shown promising efficacy in treating CIPN [76,77]. Second, high levels of fatigue are commonly associated with CIPN [32,60]. Therefore, it is reasonable to examine the hypothesis that yoga could be an effective intervention for CIPN by reducing fatigue.

There are also potential psychosocial mediators of the relationship between exercise and CIPN. For instance, depression, anxiety, and loneliness have been associated with CIPN, perhaps due to a common underlying etiology [32,60]. If clinicians can identify a patient with CIPN who may suffer from depression, anxiety, or loneliness, they may be able to recommend an exercise regimen that is primarily directed towards driving a positive psychosocial response. This may include exercise in a group setting, partner exercise, or a training class. Any of these options could build a patient’s social support network and potentially alleviate some degree of depression. To understand the role of patient expectation in this effect, it may be important to examine the interplay of exercise with placebo and nocebo effects, in which anticipation of pain relief or increase can induce hypoalgesia and hyperalgesia, respectively [78]. Future work in this area could focus on these relationships in the context of CIPN with an approach like that of Colloca et al., 2018, which concluded that pain sensations can be reduced through isotonic exercise and reinforcement of positive expectations [78].

### 4.2. Moderators of the Effects of Exercise on CIPN

We examined two past studies that conducted moderation analyses and speculated other potential moderators based on the current exercise and cancer-related literature.

Courneya et al., 2014 conducted one of the first studies to explore moderators of the effects of exercise on CIPN [20]. Women with breast cancer (*n* = 301) were randomized to supervised exercise three times a week with (a) a standard dose of 25–30-min aerobic exercise, (b) a higher dose of 50–60-min aerobic exercise, or (c) a higher dose of 50–60 min combined aerobic and resistance exercise. The primary outcomes were patient-reported physical function (i.e., assessed with subscales of the Medical Outcomes Survey Short Form (SF-36)—at baseline, twice during chemotherapy, and 3–4 weeks after) and health-related fitness parameters (i.e., aerobic fitness, muscular strength, whole body fat mass, and lean body mass). The study used the Functional Assessment of Cancer Therapy-Taxane (FACT-Taxane) to assess taxane/neuropathy symptoms as a secondary outcome. The moderators explored included a patient’s baseline demographics (i.e., age, sex, marital status, menopausal status), fitness status, body composition parameters (i.e., body mass index (BMI), baseline aerobic fitness), and cancer variables (i.e., stage of cancer, type of surgery and treatment, length of chemotherapy). BMI, menopausal status, age, and baseline aerobic fitness moderated the effect of exercise for neuropathy symptoms. The results suggest that younger patients with a higher baseline aerobic fitness level and a healthy BMI may benefit most significantly from higher-intensity aerobic and resistance training performed three times a week. At a physiological level, these results could be explained in part by functional decline and/or protracted low-grade inflammation commonly associated with advanced age and a higher BMI [79] that may impede a patient’s ability to benefit from higher exercise doses [20]. However, because this study did not include a non-exercise control group, the absolute benefits of exercise could not be determined (i.e., even patients who benefited the least might have experienced clinically meaningful improvements).

Kleckner et al., 2018 conducted another exploratory secondary analysis that provides insight into potential moderators of the effects of exercise on CIPN [21]. Patients starting chemotherapy (*n* = 355) were assigned to low-moderate intensity walking and moderate-intensity resistance band programs. They were encouraged to increase their daily steps by 5–20% each week and increase repetitions, sets, and resistance levels of the 16 resistance band exercises over the course of six weeks. CIPN severity was examined using two numerical rating scales of hot/cold sensations in the hands/feet and numbness/tingling. Exercise tended to reduce hot/cold sensations in the hands/feet and numbness/tingling compared to the control. Age, sex, and cancer type were found to moderate these effects. Specifically, exercise benefited patients who were older, male, or had breast cancer. At a physiological level, these results could be explained in part by exercise-induced reduction of inflammation and changes in the brain that counteract sensitization associated with neuropathic pain [21].

As the Courneya and Kleckner studies were both exploratory secondary analyses (i.e., the studies were not designed to assess CIPN or the moderators of exercise and CIPN) [20,21], this work sets the stage for follow-up studies using similar interventions that further explore these moderators more rigorously (i.e., using a priori hypotheses, outcome measures, and power analyses that determine the sample sizes needed to test for replication).

We can potentially learn about moderators of the effects of exercise on CIPN by reviewing studies dealing with moderators of the effects of exercise on other symptoms reported by patients with cancer. For instance, Kalter et al., 2015 identified demographic factors (e.g., age, sex), clinical factors (e.g., type of treatment, time since treatment), and psychological factors (fatigue, self-efficacy, symptoms of depression and anxiety) that moderated the effects of a 12-week group-based exercise program on the self-reported quality of life of 209 cancer survivors [80]. Groups of 8–10 patients were randomized to physical training, physical training plus cognitive behavioral therapy, or a waitlist control group. The results revealed that exercise worked best for the participants who received radiotherapy, particularly those who received chemoradiotherapy, as opposed to those who did not. The patients with high baseline levels of fatigue experienced greater benefit than those with low baseline levels of fatigue. While these results were intended to indicate moderators of the effect of exercise on patients with cancer, they may also indicate a moderator of the effect of exercise on patients with CIPN. Higher levels of fatigue have been associated with worse CIPN [32,60], so patients with CIPN and high baseline fatigue may have the greatest reduction in CIPN. However, because radiotherapy and chemotherapy will vary depending on cancer cohort, these results may not be generalizable.

It is also possible to theorize the effects of moderators on exercise and CIPN based on the related findings in the current relevant literature. Various studies have illustrated that age may moderate the effect of exercise on CIPN symptoms, including the aforementioned Courneya et al., 2014 and Kleckner et al., 2018 studies. Courneya et al., 2014 found that younger patients were more likely to benefit from a high volume of aerobic exercise than older patients, while older patients required less exercise to mitigate CIPN symptoms than younger patients [20]. The results of Kleckner et al., 2018 are consistent with this idea, suggesting that low-to-moderate aerobic and resistance exercise treats CIPN better in older patients [21]. A third study connects age to a related moderator such as fear of falling. Schwenk et al., 2016 investigated the effect of an interactive motor adaptation balance training program on improving balance in patients with CIPN (≥ 55 years) [81]. Twenty-two patients were randomized to either complete a balance training program or receive no intervention over the course of four weeks. The outcome measures were (1) balance and gait performance, which were assessed using wearable sensors, and (2) fear of falling, which was evaluated with the Falls Efficacy Scale-International (i.e., a 16-question survey that assesses a patient’s fear of falling during physical and social activities). The study concluded that the participants with a higher fear of falling at baseline had a better training response [81]. 

Collectively, the results of these studies indicate that older patients with a higher fear of falling may be more prone to responding positively to exercise, especially balance training. This is consistent with other research showing that by emphasizing proprioceptive information in patients, balance training has been shown to improve walking, gait, and balance and also reduce the risk of falls [56]. By identifying a specific moderator of response (i.e., age and fear of falling), predictions can be made to determine which subgroups of patients would respond best to specific types of exercise. Further research on the moderators of exercise and CIPN is necessary to inform accurate predictions and recommendations. Ultimately, this knowledge would allow healthcare providers to better understand patients and prescribe the most effective treatments based on a patient’s individual characteristics.

## 5. Biomarkers

A biomarker is a quantitative measurement that correlates with a biological process in a healthy or pathogenic state [82]. In the context of mediators and moderators, a biomarker can be considered a measurement on mechanistic pathways (i.e., a mediator) [83]. Leveraging biomarkers can allow clinicians to gain a holistic assessment of CIPN and tailor treatment to patients according to baseline characteristics and risks. From a clinical trial design perspective, biomarkers are valuable because they are objective measurements of a patient’s condition (i.e., biomarkers are not subject to behavioral artifacts). Biomarkers can guide the design of randomized clinical trials to evaluate biomarker-guided therapy with different patient subgroups and different recommended management [83]. Additionally, if the primary outcome of a clinical trial is null, biomarkers can suggest whether the tested intervention in fact targeted the expected mechanism of action [82]. Although there has been substantial work on developing salient biomarkers of CIPN severity and progression, no biomarkers have yet been implemented for routine clinical use. We identified promising biomarkers for CIPN that are relevant to exercise based on preclinical models and clinical studies of CIPN, exercise, and conditions related to CIPN (i.e., diabetic neuropathy, chronic pain). These biomarkers can inform predictions of who will respond best to exercise and which symptoms are best targeted by specific exercises.

We summarized details for four classes of exercise CIPN biomarkers in Table 1, Table 2 and Table 3 and provide a brief summary below. First, the most promising biomarkers are related to inflammation, including cytokines IL-1β and IL-10. IL-1β is a proinflammatory cytokine increased during chemotherapy that contributes to CIPN development [31]; levels of IL-1β have been shown to decrease after exercise [84]. IL-10 is an anti-inflammatory cytokine that reduces CIPN-associated inflammation; levels of IL-10 have been shown to increase after exercise [33]. Therefore, inflammatory cytokine levels could form a biomarker or set of biomarkers for the effects of exercise on CIPN.

Second, BDNF is another promising biomarker of exercise’s effect on CIPN based on clinical studies. Higher levels of BDNF have been associated with lower severity CIPN symptoms [89], while lower levels of BDNF have been associated with higher severity neuropathy [88]. BDNF levels have also been shown to increase significantly during exercise [98]. Therefore, BDNF levels could be a biomarker for susceptibility to CIPN [89].

Third, biomarkers using brain imaging (e.g., brain hyperactivity and hyperconnectivity using functional MRI) may be useful to characterize the relationship between exercise and CIPN. Various clinical studies support the idea that exercise may have different effects on the brain based on an individual’s clinical condition. Additionally, the magnitude of the effects of exercise on the brain may depend on the severity of the condition (i.e., the brains of patients with mild cognitive impairment versus moderate-to-severe condition, etc., respond differently to exercise) [100,117]. In patients with chronic pain conditions, brain markers, such as functional connectivity, show sensitivity to exercise-induced change [101,117], which indicates that they may be viable biomarkers for future study in CIPN.

Fourth, the evaluation and measurement of mitochondrial state and activity may also provide valuable biomarkers for the effect of exercise on CIPN. There are various established biomarkers of mitochondrial function based on clinical studies, including citrate synthase activity, that could be applied to understand how exercise stimulates the mitochondria and how it affects CIPN [103]. Other more speculative factors based on in vivo and in vitro models may also be important in identifying mitochondrial biomarkers. For instance, because exercise is known to trigger mitochondrial biogenesis, quantifiable rates of mitophagy and biogenesis could be indicators of the effect of exercise [118].

In addition, there is emerging literature and interest in the neurofilament light chain (NfL) as a biomarker of axonal degeneration in CIPN. NfL is a general marker of axonal degeneration relevant across neurological disorders, and a recent study found evidence that it may be responsive to exercise for patients with multiple sclerosis (MS). The study examined the effect of aerobic exercise on NfL levels in MS patients randomized to an exercise study group (three times a week at 60–70% of maximal aerobic capacity + home exercise) or a control group (at-home exercise program) over the course of eight weeks. The results reported a significant reduction of NfL in the exercise study group compared to the control group, suggesting that NfL may be a promising biomarker to understand the regulation of disease activity via exercise in MS patients and could be viable for future study with exercise and CIPN.

## 6. Clinical Implications

In the near future, knowledge of mediators and moderators could help advance the prevention and treatment of CIPN with exercise. Clinicians would better understand (1) for whom and under what conditions exercise works best and (2) how to optimize exercise for individual patients, as has been suggested for research in treating psychiatric conditions [119].

Mechanistic knowledge gained from the study of mediators and moderators can improve CIPN prevention techniques. Psychosocial factors (e.g., motivation and expectations, other symptoms) can work in conjunction with biological information to target risk factors for CIPN [17]. For example, high levels of inflammation (e.g., cytokines IL-1β) in patients undergoing chemotherapy have been implicated as a risk factor for CIPN [32]. In certain cases, a patient known to have high inflammation prechemotherapy could be prescribed with an exercise program designed to decrease levels of proinflammatory IL-1β and increase levels of anti-inflammatory IL-10. Understanding this benefit may, in turn, motivate the patient to adhere to an exercise regimen that could decrease personal risk of CIPN.

Prescribing exercise as a treatment for CIPN is an individually based process [16], and in the future, we envision a workflow that leverages knowledge of mediators and moderators of the effects of exercise on CIPN. A clinical care team would first examine the patient’s medical history, specifically potential comorbidities and concurrent medications, to guide the development of a personalized exercise program (e.g., prior exercise experience, physical fitness, group-based exercise may be helpful for patients experiencing loneliness). Second, clinical tests (e.g., bloodwork, sensory testing of neuropathy symptoms, assessment of functional limitation and balance/gait) would provide a clearer picture of a patient’s specific condition and CIPN symptoms so that clinicians could evaluate which exercise prescriptions might be most effective for that patient (e.g., specific hand exercise for hand cramps, specific balance exercise for sensory loss in the feet) [16] according to the guidelines for the clinical implementation of exercise provided by the 2018 American College of Sports Medicine Roundtable [17]. In particular, sensorimotor training appears to be a crucial factor of exercise-induced improvement to CIPN, which justifies its consideration in this process [18]. Third, an interview could reveal information about patient perspective and goals that could guide decisions regarding specific exercise preference (e.g., a patient with a high risk of falling may prefer seated exercises) because patients who enjoy specific exercises are more likely to adhere in the long term and achieve sustained positive results. Clinicians would be able to determine how significant the benefit of exercise would be for a patient and make recommendations accordingly.

## 7. Summary and Future Work

Although exercise is not currently recommended in clinical guidelines to treat CIPN due to lack of definitive phase III clinical trials, there are approximately two dozen published clinical trials of exercise and CIPN; about a dozen randomized controlled trials, the majority of which suggest its efficacy [15]; and a recent meta-analysis estimating small-to-medium beneficial effects [18]. It is plausible that exercise will soon be shown to be beneficial for CIPN. The next steps for this field are to develop full mechanistic understanding of the effects of exercise to determine how it can be optimized to best treat CIPN symptoms and designed for patients who are most prone to respond. Due to the highly limited knowledge in this area, the goal of our review was to approach the study of how exercise may treat CIPN through a novel lens by studying mediators, moderators, and biomarkers at the neurophysiological and psychosocial levels. To expand this investigation, we propose several avenues for future research.

First, we need more definitive knowledge on how exercise exerts its effects at the neurophysiological level (axonal integrity and outgrowth, neurotrophic factors, inflammation, mitochondrial function, and the brain) and from the psychosocial perspective (mood, social support and self-efficacy, and expectation of benefit). This understanding can be developed through work with (1) preclinical models, which would enable more detailed and rigorous mechanistic processes, and (2) clinical trials in humans, which would provide a clearer picture of real-world applications. Ideally, these studies would also systematically examine the effects of exercise on patients undergoing different types of chemotherapy, considering the evidence that the mechanisms through which exercise impacts CIPN may vary for the specific toxicity profiles of different chemotherapy types (e.g., platinum vs. taxane) [2]. Because of this heterogeneity, further study of toxicity profile across various classes of chemotherapy could offer insight into the etiology of CIPN and, ultimately, the relationship between exercise and CIPN. Currently, there are not enough studies to make inferences about which chemotherapy types exercise works best for and whether the mechanism of action of exercise differs by chemotherapy type. Additionally, it would be beneficial for studies to consider the different inflammatory effects of acute versus chronic exercise [120] on the development and experience of CIPN as acute and chronic exercise have different effects on inflammation [120] and other outcomes [121]. Both factors are critical in understanding how exercise will affect a patient at a neurophysiological level. This knowledge can ultimately allow for the tailoring of exercise regimens to patients and the maximization of adherence and benefit. It may also be possible to learn about the mechanistic pathways of exercise by examining null pharmacological trials. For example, due to its beneficial effects on mitochondrial function and the role of mitochondrial function in CIPN, the compound acetyl-L-carnitine was believed to be a mechanistically sound treatment for CIPN based on preclinical evidence. However, it was found to have harmful effects when tested in phase III clinical trials [12,13]. Although exercise also has mitogenic effects, these mechanisms may differ from the effects of acetyl-L-carnitine if exercise proves to be beneficial as acetyl-L-carnitine was shown to be harmful for CIPN. Further analysis of this outcome could elucidate the role of exercise in treating CIPN. This insight could also offer benefits beyond this area of study by informing the discovery of other interventions that prevent or treat CIPN through similar pathways.

Second, we need to develop a much deeper understanding of which mediators and moderators are most relevant in the context of exercise and CIPN. To rigorously advance this body of research, we believe this question should be investigated with tightly controlled clinical trials (e.g., at academic medical centers) and pragmatic real-world trials (e.g., at a variety of community sites) specifically designed to test one or more potential moderators or mediators of the effects of exercise on CIPN. We recommend that this testing include the mediators and moderators noted in this review (i.e., inflammation and self-efficacy as the mediators to explain how exercise exerts it effects; body mass index and age as the moderators to predict who will respond best to exercise). The analyses for these studies should include a large sample size that accurately reflects the broader patient population. In addition, studies should also be designed to address health disparities for underrepresented groups (e.g., racial or ethnic groups, low-income households, patients living in rural areas) so care can be advanced for patients from diverse backgrounds [122]. This would include addressing any specific barriers for those groups (e.g., access to exercise) and considering ways to optimize the benefits of exercise (e.g., treating diabetes concurrent with CIPN) [122]. Such barriers would best be removed by working with community stakeholders from underrepresented groups to inform methods of reaching individual patients most effectively.

Third, it is essential to discern how the identification of mediators and moderators can inform the implementation of exercise into clinical settings. Ideally, mediators will inform tailoring and optimizing exercise prescriptions for specific CIPN phenotypes. Moderators will aid predictions of patient response to different types of exercise (e.g., resistance, endurance, and sensorimotor training), which may play an important role in determining the degree of benefit, as suggested by a recent meta-analysis of the effects of exercise on CIPN [18]. We recommend that well-designed clinical trials be designed to evaluate the extent to which mediators and moderators will be useful for clinicians and patients.

## 8. Conclusions

Exercise for the treatment and prevention of CIPN is a rapidly growing area of research, and as supporting work continues to be published, the next step for this field is to delve deeper into the mechanisms, mediators, and moderators that describe how exercise exerts its benefits for patients. We are optimistic for this trajectory of work and hope that emerging programs of study will continue to corroborate and expand upon current mechanistic knowledge, identify the most useful mediators and moderators, and further understanding of how to best apply these findings in the real world to ultimately reduce the burden of CIPN and chemotherapy on patients with cancer.

## Figures and Tables

**Table 1:** Potential biomarkers in relation to CIPN.

Biomarker	Citation	Population	Methods	Results
Inflammation	Kleckner et al., 2021 [32]	116 women with breast cancer	- Predicted CIPN severity at 6 weeks using prechemotherapy assessments of inflammatory factors	- Higher levels of proinflammatory cytokines IFNγ and IL-1β and lower levels of anti-inflammatory IL-10 predicted numbness/tingling and hot/coldness in the hands and feet
Inflammation	Bujalska et al., 2008 and 2009 [85,86]	Rat model of chemo- and diabetic neuropathy	- Neuropathy induced with vincristine (VIN) administration through the tail vein for 10 days (70 µg/kg solution with VIN sulfate and distilled water) - Changes in nociceptive thresholds determined using mechanical stimuli before drug administration and after drug discontinuation	- Select bradykinin antagonists (i.e., B2 or B1) prevented inflammation and development of VIN-induced hyperalgesia - Activation of bradykinin receptor inhibitors and cox pathways, which can cause inflammation, may play an important role in the development of CIPN pain
Inflammation	Leitzelar et al., 2021 [87]	Review article	- Narrative review - Literature search for observational and experimental studies of exercise and neuropathic pain in Academic Search Premier, Google Scholar, PubMed, and Web of Science - Search concluded on 02 June 2020	- Proinflammatory markers (e.g., TNF-α, IL-1β, IL-6) are released in response to microglial activation after nerve damage in CIPN - Elevated after acute exercise but reduced after chronic exercise - Exercises influences pain through inflammatory marker changes
Brain-derived neurotrophic factor (BDNF)	Szudy-Szczyrek et al., 2020 [88]	91 patients with multiple myeloma	- Detection of BDNF in serum with ELSA - Neuropathy assessed according to CTCAE v5	- Positive correlation between the serum BDNF concentration before treatment and the severity of polyneuropathy - BDNF showed significant diagnostic usefulness in the diagnosis of CIPN
BDNF	Azoulay et al., 2019 [89]	45 patients with multiple myeloma and non-Hodgkin lymphoma with CIPN	- Measured objective CIPN signs (Total Neuropathy Scale-Revised) and subjective CIPN symptoms - BDNF protein levels and the Val66Met SNP determined with ELISA and Sanger sequencing	- Higher baseline serum BDNF levels were related to the development of lower objective CIPN signs and subjective CIPN symptoms - BDNF was a useful marker to predict patient response after treatment and showed significant usefulness in diagnosis of CIPN
BDNF	Cavaletti et al., 2004 [90]	62 women with squamous cervical carcinoma	- Examined and scored patients’ CIPN according to TNS before and after chemotherapy	- Reduced levels of BDNF family member, NGF (nerve growth factor) were found to be associated with CIPN development and severity
Brain structure and function	Nudelman et al., 2016 [91]	47 patients with nonmetastatic breast cancer	- Examined progression of CIPN symptom severity before treatment, a month after completion, and a year after completion	- At one month, CIPN severity was associated with greater perfusion in superior frontal gyrus, cingulate gyrus, left middle gyrus, medial frontal gyrus - From prior to chemotherapy to one month after chemotherapy, decreases in CIPN severity and perfusion were associated with decreased gray matter density in the left middle/superior frontal gyrus - At one year, there were no significant associations between CIPN severity and brain perfusion
Brain structure and function	Boland et al., 2014 [38]	12 patients with multiple myeloma or CIPN and 12 healthy volunteers	- Neurophysiological and clinical assessments: peripheral nerve conduction studies, sensory testing, and TNS-reduced version - Chronic pain acceptance questionnaire - Heat stimulation protocol - fMRI scanning protocol	- Patients exhibited greater fMRI BOLD activation in the left precuneus and lower activation in the right superior frontal gyrus for the foot and thigh (7/10 pain rating) in comparison to the volunteers - Activation in the left front operculum in response to heat pain stimulation of the foot was associated with worse CIPN
Brain structure and function	Prinsloo et al., 2017 [92]	62 cancer survivors with CIPN	- Randomized 30 participants to EEG neurofeedback, 32—to waitlist control	- Neurofeedback increased alpha power, decreased beta power, reduced worst pain, average pain, and pain features (e.g., unpleasantness) in experimental group compared to waitlist control
Mitochondria	Agalave et al., 2021 [93]	3 male mice and 3 female mice in multiple experimental groups	- Measured mitochondrial energy provision efficiency and bioenergetics on murine dorsal root ganglia neurons via extracellular flux (“Seahorse”) with paclitaxel treatment	- Examined the importance of eIF4E phosphorylation in the development of CIPN - Higher basal and nonmitochondrial respiration in males - Higher ATP turnover and maximal respiratory capacity in females
Mitochondria	Khasabova et al., 2019 [94]	Murine model of cisplatin-induced hyperalgesia	- Prepared primary cultures of dorsal root ganglion neurons - Observed cisplatin-increased oxidative stress measured using the oxidation of chloromethyl-29,79-dichlorodihydrofluorescein diacetate - Active mitochondria were assessed in ex vivo dorsal root ganglia neurons using a membrane-permeable probe sensitive to the inner mitochondrial membrane potential (Mito Tracker Deep Red FM)	- Noticeable decrease in antioxidant enzyme activity (catalase and SOD) - Incubation of freshly cultured neurons with cisplatin reduced the relative density of Mito Tracker-labeled mitochondria - Cisplatin-increased oxidative stress increased the sensitization of the neurons
Mitochondria	Zheng et al., 2012 [95]	Mouse model of bortezomib-induced peripheral neuropathy	- Investigated mitochondrial bioenergetics in isolated sciatic nerves - Assessed oxygen consumption before and after additions of glutamate + malate, ADP, rotenone, succinate, and cytochrome c	- Found bortezomib-induced decreases in complex I- and complex II-mediated respiration - Noted reduction in ATP production using the same oxygen consumption chamber but with additions of ADP + glutamate + malate + succinate

**Table 2:** Potential biomarkers in relation to exercise with healthy individuals or CIPN-related conditions.

Biomarker	Citation	Population	Methods	Results
Inflammation	Gleeson et al., 2011 [33]	Review article of healthy and patient populations	- Narrative review of mechanisms through which acute and chronic exercise has anti-inflammatory effects and implications of these effects for prevention and treatment of disease	Exercise has potent anti-inflammatory effects on the body by: (1) Promoting the release of IL-6 into circulation from contracting muscles (2) Increasing circulating levels of IL-10 and IL-1 receptor antagonist (3) Increasing circulating numbers of IL-10-secreting regulatory T cells (4) Inhibiting proinflammatory cytokine production, antigen presentation, and costimulatory molecule expression (5) Reducing numbers of proinflammatory cytokine-producing monocytes
Inflammation	Kleckner et al., 2019 [36]	293 patients with cancer undergoing chemotherapy Exploratory/hypothesis-generating secondary analysis of a randomized clinical trial	- Randomized controlled trial - 6-week walking and resistance exercise program during chemotherapy vs. usual care - Exploratory assessment of serum concentrations of cytokines pre- and post-intervention	- Exercise strengthened the correlation between concentration changes of IL-10 and IL-6, IL-10 and IL-1β, and IL-10 and TNFR1 - Exercise reduced proinflammatory cytokines IFNγ and possibly IL-1β
Inflammation	Parent-Roberge et al., 2020 [96]	20 non-metastatic cancer patients initiating chemotherapy and/or hormone therapy	- Randomized controlled trial - 12 weeks of supervised, combined exercise vs. control group (static stretching) - Primary outcomes: inflammatory profile - Functional Assessment of Chronic Illness - Therapy–Fatigue questionnaire	- Exercise improved the IL-6/IL-10 ratio during chemotherapy for nonmetastatic cancer patients
Inflammation	Schauer et al., 2021 [97]	Secondary analysis of a randomized controlled trial *n* = 600 breast, prostate, colorectal cancer patients undergoing primary adjuvant cancer treatment	- Six months of high-intensity (HI) vs. low–moderate (LMI)-intensity combined aerobic and resistance exercise during and after treatment - Plasma samples taken at baseline, post-treatment, post-intervention and analyzed for inflammatory cytokines, TNF-α, CRP	- HI exercise during chemotherapy reduced inflammation more than LMI exercise - HI exercise yielded a smaller increase in inflammation after chemotherapy vs. LMI exercise - HI exercise resulted in lesser increases in CRP and TNF-α immediately post-treatment compared to LMI exercise
Brain derived neurotrophic factor (BDNF)	Szuhany et al., 2015 [98]	Meta-analysis focusing on 29 studies (*n* = 1111 participants in total in healthy, multiple sclerosis, mild cognitive impairment, or major depressive disorder populations)	- Examined the effect of exercise on BDNF levels in three exercise paradigms: (1) single session of exercise, (2) session of exercise following a program of regular exercise, (3) resting BDNF levels following a program of exercise	- Moderate effect of increased BDNF was found after one session of exercise - Regular exercise intensified this effect - Small effect of increased resting BDNF levels after regular exercise - Men’s BDNF levels changed more than females’ levels in response (sex as a moderator)
BDNF	Smoak et al., 2021 [99]	Correlational study *n* = 32 participants either receiving chemotherapy and/or radiotherapy or not receiving therapy	- Conducted cardiorespiratory fitness, muscular strength, depression, fatigue, and quality of life assessments for 7 days	- BDNF was positively related to light physical activity outside of exercise training for patients receiving treatment
Brain structure and function	Ellingson et al., 2016 [100]	Case–control correlational + crossover interventional study with acute exercise and a control condition (quiet rest) *n* = 9 fibromyalgia (FM) patients and *n* = 9 non-FM controls	- Two functional neuroimaging scans following exercise and following quiet rest - Brain response and pain ratings to noxious heat stimuli compared within/between groups	- Exercise decreased pain sensitivity in FM patients - Appeared to stimulate brain regions involved in descending pain inhibition in FM patients - FM patients showed greater activity in brain regions involved in pain modulation (e.g., the dorsolateral prefrontal cortex) following exercise in comparison to the control participants
Brain structure and function	Voss et al., 2010 [101]	Randomized controlled trial *n* = 97 older adults and younger adults (older adults: 55 < x < 80; younger adults: 18 < x < 35)	- One-year randomized intervention trial to compare the effects of aerobic vs. nonaerobic fitness training on brain function	- Increased functional connectivity between aspects of the frontal, posterior, and temporal cortices - Effects of exercise were greater in older adults compared to younger adults (age as a moderator)
Mitochondria	Cao et al., 2012 [102]	Randomized preclinical experimental study *n* = 50 male mice	- Randomized to a control group or to participate in 10, 30, 60, 90 min of swimming per day	- Habitual exercise increased the mitochondrial DNA (mtDNA) copy number in the gastrocnemius muscle of mice
Mitochondria	Vigelsø et al., 2014 [103]	Review article	- Integrated 93 human studies from 70 publications	- Supports citrate synthase activity as a biomarker of mitochondrial function in skeletal muscle - Concluded a positive correlation between change in citrate synthase activity and training adaptations, including maximal oxygen uptake (VO_2_max) in skeletal muscle
Mitochondria	Mijwel et al., 2018 [104]	Randomized controlled trial *n* = 23 women with breast cancer	- Obtained resting skeletal muscle biopsies pre- and post-intervention: 16 weeks of high-intensity interval training (HIIT), resistance training + HIIT, or usual care	- Exercise increased the mitochondrial content in skeletal muscle among patients with breast cancer as assessed by citrate synthase activity
Mitochondria	Balan et al., 2019 (for review see Bo et al., 2020) [105,106]	Cohort correlational study of 33 young sedentary, old sedentary, young active, and old active men	- Markers for mitophagy, fission, fusion, mitogenesis, mitochondrial metabolism were assessed with qRT-PCR, Western blotting, immunofluorescence staining	- Rates of mitochondrial fusion and fission can be quantified using Western blotting of proteins and/or qRT-PCR of mRNA transcripts (e.g., Mito fusion 1 (MFN1), Mito fusion 2 (MFN2), dynamin-related protein 1 (DRP1), mitochondrial fission 1 (FIS1)) - Chronic exercise behavior is associated with increased rates of fusion and fission, but not always

**Table 3:** Potential biomarkers in CIPN-related conditions.

Biomarker	Citation	Population	Methods	Results
Inflammation	Purohit et al., 2021 [107]	Case–control correlational study *n* = 694 type 1 diabetes patients (*n* = 507 patients without peripheral neuropathy (nDPN) and *n* = 187 patients with peripheral neuropathy)	- Measured soluble cytokine receptors, markers of systematic and vascular inflammation (e.g., IL-1RA, IL-8) using multiplex immunoassays - Ridge regression used to create a multilevel protein score that accounted for serum levels of different proteins	- Serum levels were elevated in DPN patients independent of sex, age, duration of diabetes - Activation of inflammatory pathways in DPN patients could be a clinical tool to identify type 1 diabetes patients for treatment with anti-inflammatory therapies
Inflammation	Cameron et al., 2008 [108]	Review article	- Evaluated the animal models which begin to establish the nuclear factor (NF)-κB cascade as a therapeutic target for neuropathy	- Activation of an inflammatory NF-κB cascade is central to etiology of diabetic neuropathy - Many drugs developed to treat diabetic neuropathy suppress NF-κB or the production of cytokines like TNF-α that stimulate NF-κB
Inflammation	Vendrell et al., 2015 [109]	Review article	- Reviewed the importance of cytokines in cancer pain - Discussed strategies to control cancer pain	- Production and secretion of proinflammatory cytokine IL-1β is associated with pain in tumor growth - Potential contributor to the genesis of neuropathic pain - IL-6 serum levels contribute to development of neuropathic pain behavior - TNF-α initiates activation of other cytokines and growth factors during an inflammatory response - Schwann cells can produce TNF-α and are therefore theorized to have an important role in neuropathic pain
Brain-derived neurotrophic factor (BDNF)	Nitta et al., 2002 [110]	Randomized preclinical interventional study Rats with diabetes induced by streptozotocin	- Measured neuronal cytoskeleton proteins calbindin, synaptophysin, syntaxin - Morphological observation by Golgi staining - Measured the content of BDNF in the brains of rats	- Found that BDNF levels were severely reduced in diabetic brains in comparison to nondiabetic brains - Synapse dysfunction associated with diabetic neuropathy is caused in part by failure to produce adequate BDNF levels in the brain
Brain-derived neurotrophic factor (BDNF)	Ge et al., 2019 [111]	Randomized preclinical interventional study Rats with comorbid diabetic neuropathic pain (DNP) and depression (DP)	- Changes in pain-related behaviors were monitored by thermal withdrawal latency and mechanical withdrawal threshold - Quantitative real-time PCR and Western blotting to detect mRNA and protein expression levels of BDNF in rats	- Upregulation of BDNF concentrations causes an antidepressant effect - Downregulation of BDNF accounts for depression - BDNF is confirmed by various studies as an important target for depression because increased levels of BDNF in the hippocampus mediate the effects of antidepressants
Brain structure and function	Harte et al., 2016 [112]	Randomized controlled trial/case–control correlational study *n* = 17 patients with fibromyalgia (FM) and *n* = 17 healthy controls	- Participants underwent aversive visual stimulation during fMRI imaging before/after receiving pregabalin treatment/placebo	- FM patients—the pregabalin treatment resulted in a decrease in bilateral anterior insular activation compared to the healthy controls - Anterior insula may be a multisensory integration site
Brain structure and function	Van der Miesen et al., 2019 [113]	Review article	- Searched for articles on neuroimaging-based biomarkers for pain on PubMed through 31 December 2018	- Structural MRI has been used to characterize and predict the incidence of chronic visceral pain, musculoskeletal pain, migraine pain - Secondary somatosensory cortex and motor regions can help to distinguish between patients with chronic pain conditions and healthy patients - Chronic pain has been associated with elevated EEG frequency energy and reduced alpha energy
Brain structure and function	Omran et al., 2021 [39]	Review article	- Searched for articles on interventional studies and correlational studies of the brain and CIPN on PubMed	- CIPN is associated with brain hyperactivity, reduced GABAergic inhibition, neuroinflammation, and overactivation of GPCR/MAPK pathways - Observed in the thalamus, periaqueductal gray, anterior cingulate cortex, somatosensory cortex, and insula
Mitochondria	Ascensao et al., 2021 [114]	Review article	- Aimed to analyze the effect of physical exercise in cardiac tolerance of animals treated with acute + sub-chronic does of doxorubicin	- There are ways to measure oxidative stress on both cellular and systemic levels - One can quantify DNA damage (8-hydroxydeoxyguanosine), lipid peroxidation (e.g., malondialdehyde), relative levels of reduced to oxidized glutathione (GSH:GSSG), or antioxidant enzyme activity (e.g., superoxide dismutase (SOD) SOD2 is compartmentalized in the mitochondria)
Mitochondria	Filler et al., 2014 [115]	Review article	- Searched PubMed, Scopus, Web of Science, and Embase databases for studies that investigated the association of markers of mitochondrial dysfunction with fatigue	- Serum coenzyme Q10 concentrations correlate negatively with fatigue in chronic fatigue conditions such as cancer-related fatigue - Coenzyme Q10 is a fat-soluble compound that carries electrons from complex II to complex III in the electron transport chain
Mitochondria	Fabbri et al., 2017 [116]	248 participants without diabetes	- Assessed mitochondrial capacity in skeletal muscle as postexercise phosphocreatine recovery time constant (τPCr) by 31P-magnetic resonance spectroscopy	- Correlated the post-exercise phosphocreatine recovery time with insulin resistance

Abbreviations: BDNF—brain-derived neurotrophic factor, CIPN—chemotherapy-induced peripheral neuropathy, CRP-C—reactive protein, EEG—electroencephalography, FM—fibromyalgia, GABA—γ-aminobutyric acid, GPCR-G—protein-coupled receptors, GSH—glutathione (reduced form), HI—high intensity, IFN—interferon, IL—interleukin, IL-1RA—interleukin 1 receptor agonist, LMI—low-to-moderate intensity, MAPK—mitogen-activated protein kinase, mtDNA—mitochondrial DNA, NF-κB—nuclear factor κ-light-chain-enhancer of activated B cells, SOD—superoxide dismutase, TNF—tumor necrosis factor, TNFR1—tumor necrosis factor receptor 1.
